# Altered brain networks and connections in chronic heart failure patients complicated with cognitive impairment

**DOI:** 10.3389/fnagi.2023.1153496

**Published:** 2023-04-14

**Authors:** Meixia Wang, Bo Xu, Xiaoxia Hou, Qianru Shi, Huimin Zhao, Qian Gui, Guanhui Wu, Xiaofeng Dong, Qinrong Xu, Mingqiang Shen, Qingzhang Cheng, Hongxuan Feng

**Affiliations:** Department of Neurology, Gusu College of Nanjing Medical University, Affiliated Suzhou Hospital of Nanjing Medical University (Suzhou Municipal Hospital), Suzhou, China

**Keywords:** chronic heart failure, cognitive impairment, DTI, graph theory, rich-club

## Abstract

**Objective:**

Accumulating evidence shows that cognitive impairment (CI) in chronic heart failure (CHF) patients is related to brain network dysfunction. This study investigated brain network structure and rich-club organization in chronic heart failure patients with cognitive impairment based on graph analysis of diffusion tensor imaging data.

**Methods:**

The brain structure networks of 30 CHF patients without CI and 30 CHF patients with CI were constructed. Using graph theory analysis and rich-club analysis, changes in global and local characteristics of the subjects’ brain network and rich-club organization were quantitatively calculated, and the correlation with cognitive function was analyzed.

**Results:**

Compared to the CHF patients in the group without CI group, the CHF patients in the group with CI group had lower global efficiency, local efficiency, clustering coefficient, the small-world attribute, and increased shortest path length. The CHF patients with CI group showed lower nodal degree centrality in the fusiform gyrus on the right (FFG.R) and nodal efficiency in the orbital superior frontal gyrus on the left (ORB sup. L), the orbital inferior frontal gyrus on the left (ORB inf. L), and the posterior cingulate gyrus on the right (PCG.R) compared with CHF patients without CI group. The CHF patients with CI group showed a smaller fiber number of edges in specific regions. In CHF patients with CI, global efficiency, local efficiency and the connected edge of the orbital superior frontal gyrus on the right (ORB sup. R) to the orbital middle frontal gyrus on the right (ORB mid. R) were positively correlated with Visuospatial/Executive function. The connected edge of the orbital superior frontal gyrus on the right to the orbital inferior frontal gyrus on the right (ORB inf. R) is positively correlated to attention/calculation. Compared with the CHF patients without CI group, the connection strength of feeder connection and local connection in CHF patients with CI group was significantly reduced, although the strength of rich-club connection in CHF patients complicated with CI group was decreased compared with the control, there was no statistical difference. In addition, the rich-club connection strength was related to the orientation (direction force) of the Montreal cognitive assessment (MoCA) scale, and the feeder and local connection strength was related to Visuospatial/Executive function of MoCA scale in the CHF patients with CI.

**Conclusion:**

Chronic heart failure patients with CI exhibited lower global and local brain network properties, reduced white matter fiber connectivity, as well as a decreased strength in local and feeder connections in key brain regions. The disrupted brain network characteristics and connectivity was associated with cognitive impairment in CHF patients. Our findings suggest that impaired brain network properties and decreased connectivity, a feature of progressive disruption of brain networks, predict the development of cognitive impairment in patients with chronic heart failure.

## 1. Introduction

As the organ with the highest energy consumption in the human body, the brain accounts for 20% of the human body’s resting metabolic rate (Phillips et al., 2016). Maintaining normal brain function requires a constant supply of metabolites, which is dependent on proper functioning of the heart highly. As a systemic disease, chronic heart failure (CHF) not only affects itself, but also affects other organs, including the brain (Kim and Kim, 2015). Gradual decline in cerebral blood flow (CBF) can cause chronic brain damage, resulting in cognitive impairment (CI) (Iadecola, 2017). A previous study published in the Lancet proposed the concept of cardiogenic dementia (Dementia, 1977). Some studies showed that the incidence of cognitive impairment in heart failure patients is 25–50% (Frey et al., 2021).

In recent years, the role of “bidirectional heart-brain interconnection” in the progression and outcome of chronic heart failure has gradually attracted attention (Frey et al., 2018). However, the pathogenesis of chronic heart failure complicated with cognitive impairment is still unclear. One of the possible mechanisms is that chronic regional brain tissue hypoperfusion leading to functional decline in critical brain areas (Zou et al., 2018). Cognitive function involves various aspects such as attention, immediate and delayed memory, learning, speech/language, executive function and visual/spatial/constructional. However, CHF patients complicated with CI have various aspects of cognitive impairment (Kim and Kim, 2015). Some researchers have documented neuroimaging alterations in cognition-related brain regions in CHF patients, such as varying degrees of atrophy in the frontal and temporal lobes (Serber et al., 2008). Some studies have shown that the structural changes in brain regions of the autonomic nervous system (primarily affecting the right hemisphere) in chronic heart failure patients (Roy et al., 2017), causing autonomic nervous system dysfunction, which further aggravates heart failure. In contrast, Jefferson et al. demonstrated through neuroimaging studies that decreased connection strength in subcortical white matter are associated with declined cardiac function independently, and the decrease in white matter connections in CHF patients may directly lead to changes in cognitive function (Jefferson et al., 2007).

Ovsenik et al. advocated the application of non-invasive, simple, available technologies to early predict or identify patients at risk for cognitive impairment due to heart failure (Ovsenik et al., 2021). Clinically, electroencephalogram (EEG) or functional Magnetic Resonance maging (fMRI) is often for detecting changes in brain function, and T1 structural imaging and Diffusion Tensor Imaging (DTI) are for detecting structural changes in gray and white matter (Xiao et al., 2016). At present, the evaluation of complex structural networks is mainly based on graph theory analysis, which converts brain networks into nodes and connections of edges, and quantifies network information through topological parameters (Mears and Pollard, 2016). A cross-sectional study based on DTI structural network found that changes in network topology parameters can well describe the abnormal structural connectivity in CHF patients with CI (Sun et al., 2014).

Exploring valid and non-invasive predictors of CHF complicated with CI will clinically dentify patients with poor prognosis and develop treatment plans for patients to improve quality of life. Few studies have investigated heart failure with cognitive impairment based on white matter fiber connectivity and overall brain network. This study mainly used DTI technology to characterize a weighted structural network, and quantify network topological parameters from the whole brain and specific brain regions, and describe the changes in cortical–subcortical connections, so as to observe the brain network’s topological characteristics in CHF patients with CI. We hope to provide support for in-depth studying the pathophysiological mechanism, diagnosis and treatment of chronic heart failure complicated with cognitive impairment.

## 2. Materials and methods

### 2.1. Subjects and data collection

Patients with clinically confirmed chronic HF enrolled in the present study were recruited randomly from Epilepsy Clinic of Suzhou Municipal Hospital from June 2018 to August 2022. The enrolled criteria were listed as follows: (a) 18 years old and above, no upper age limit; (b) a definite diagnosis of chronic heart failure [using the New York Heart Association (NYHA) I—IV]; (c) no history of mental illness and alcoholism; (d) no thyroid dysfunction, liver cirrhosis, hepatic encephalopathy, cerebrovascular disease and other diseases affecting cognitive function; (e) all subjects were informed and consented. The exclusion criteria: (a) a clear history of central nervous system damage, such as trauma, tumor, infection, carbon monoxide poisoning, demyelinating changes, etc.; (b) hearing, reading, language expression or writing disorders; (c) any implant or device impeding MRI; (d) a life expectancy less than 6 months.

The Montreal cognitive assessment (MoCA) is used to evaluate cognitive impairment (Nasreddine et al., 2005), which assesses different cognitive domains: Visuospatial/Executive functions (5 points), attention (6 points), naming (3 points), abstraction (2 points), language (3 points), delayed recall (5 points) and orientation (6 points). The maximum score is 30 and the minimum score is zero. A higher score indicates better cognition. If the patient has been educated≤12 years, 1 point will be added to the total score. Patients with total scores ≥26 were classified as normal cognitive function and total scores <26 as impaired cognitive function (Chialà et al., 2018).

General data collection includes gender, age, years of education, marital status, and smoking. Disease-related data including body mass index (BMI), NYHA functional class, N terminal pro B type natriuretic peptide (NT-proBNP), the left ventricular ejection fraction (LVEF), Low density lipoprotein (LDL), High density lipoprotein (HDL), serum creatinine and serum urea are mainly collected through patients’ medical records. The study participants consisted of 30 CHF patients clinically diagnosed with cognitive impairment (the CHF + CI group). The control group consisted of 30 age-, right-handed-, and mean years of education-matched CHF patients without CI (the CHF group).

The Ethics Committee of Affiliated Suzhou Hospital of Nanjing Medical University (Suzhou Municipal Hospital) approved this study (Ethics Review Number: K-2021-GSKY20210207). The 60 subjects were right-handed. Each person signed an informed consent form before the study.

### 2.2. Image acquisition

Neuroimaging data were acquired on a 3.0-Tesla MRI scanner equipped with an 8-channels head coil (Achieva TX, Philips Healthcare, Best, Netherlands). The scanning was performed in the supine position with the head fixed, and the subjects were awake with their eyes closed. Structural images of all subjects were obtained with T1-weighted scanning (slice thickness = 5 mm, TR/TE = 3,000/10 ms, slice gap = 1 mm) for spatial normalization of brains. The structural map was screened rapidly by imaging staff with 10 years of experience, and cases with obvious intracranial organic lesions (not found in this study) were preliminarily excluded. If subjects had obvious intracranial organic lesions, no further DTI scans were performed. In addition, all participants underwent DTI to acquire data for graphic theoretical analysis. DTI was obtained by spin-echo single-shot pulse sequences. The DTI parameters: flip angle = 90°, TR/TE = 6,100/93 ms; slice thickness = 2 mm, FOV = 238 mm × 238 mm, matrix = 256 × 256, and *b* = 1,000 s/mm2, diffusion gradients are applied in 30 different non-linear directions. Transfer and save raw DTI image files from the MR scanner to a computer. All image acquisitions were performed on the same MR scanner and scanned by trained stationary technicians.

### 2.3. Data pre-processing

The data of were preprocessed by “Pipeline for Analyzing braiN Diffusion imAges” (PANDA) in the MATLAB (Shu et al., 2011). The steps: converting the DICOM images into NIfTI format, estimating the mask of whole-brain, performing eddy current and head motions correction.

Apply an affine alignment of diffusion-weighted images to the b0 images with the FMRIB (FMRIB’s Software Library, FSL) Diffusion Toolbox (FDT, version 5.0; http://www.fmrib.ox.ac.uk/fsl) to calculate eddy current and head motion corrections.

Then we use pretreated DTI data to calculate the anisotropy fraction (FA) value of each patient and obtain FA images.

### 2.4. Construction of brain networks

Edges and nodes are two basic elements of brain network. Each subject’s white matter structural network was constructed using network node definitions, white matter fiber imaging, and network edge definitions in this study. (1) Defining network nodes: According to Automated Anatomic Labeling-90 (Tzourio-Mazoyer et al., 2002) (AAL-90) Atlas, the surface of cerebral cortex is divided into ninety brain regions (excluding cerebellum). In the brain network, each region serves as a node, and the edges refer to the white matter fiber tracts that connect these nodes. The acquired T1 images of each subject are registered with the corresponding FA images. Then, the T1 images of each subject are nonlinear registered with the Montreal Neurological Institute Space 152_T (MNI 152_T) template to obtain the corresponding nonlinear transformation relationship. Finally, the brain regions of the AAL90 atlas were converted into T1 images of each patient using the reversal of the nonlinear transformation relationship. (2) Defining network edges: The algorithm of fiber assignment by continuous tracking (FACT) is used to construct the white matter fiber bundles of each patient; the tracking of each fiber was interrupted when the deflection angle exceeds 45° or the FA value is less than 0.1. And the fiber number (FN) between the two brains is defined as the edge. Calculate the connection of each brain region, and the connection strength is the corresponding FA value, forming 90 × 90 weighted matrix, from which three-dimensional images of brain network structure can be visualized. In order to avoid false-positive results, we only keep the fiber connections that exist in more than 80% of the subjects, and then set the total number of fiber bundles between two nodes greater than or equal to three, and then consider that there is a connection between them (This edge is considered to be valid on the network when FN reaches three or more). This procedure has been proved to reduce the risk of false-positives and has been used in previous studies (Shu et al., 2011).

### 2.5. Calculation of brain network parameters

(1) The calculation and statistics of the whole-brain and local-regional graph-theoretic indicators were performed using Gretna software of the MATLAB platform. The brain network’s parameters mainly include:
① Global efficiency (E_glob_, E_g_) is a parameter reflecting the information transmission and integration capability of the whole brain network. *N* nodes exist in the network G. The shortest path L_ij_ is between node i and node j. The formula:
$$E_{glob}=\left[1/n\left(n-1\right)\right]\times \underset{i\neq j\in G}{\sum }1/L_{ij}$$
② Local efficiency (E_loc_) is the global efficiency of each sub-network (G_i_). It represents the local connectivity and fault tolerance. The formula:
$$E_{\text{loc}}=\left(1/n\right)\times \underset{i\in G}{\sum }E_{\text{glob}}\left(G_{i}\right)$$
③ Clustering coefficient (C_p_) indicates the possibility of nodes connecting to the other surrounding nodes, indicating the local interconnection. The formula:
$$C_{p}=(1/n)\times \Sigma \left\{\underset{i\in G}{\sum }\left[2/k_{i}(k_{i}-1)\underset{j,k}{\sum }{\left(\omega_{\text{ij}}\omega_{\text{jk}}\omega_{\text{ki}}\right)}^{\frac{1}{3}}\right]\right\}$$

K_i_ is the node I’s degree value, and the calculation method is “k”_ “i” “=“∑_ “j” “C” _ “Ij” (where C_ij_ means the connection status between nodes i and j). When node j and node k are directly connected with node i, ω represents the weight value between the two nodes.

④ Characteristic path length (L_p_) is the average of all shortest paths between all nodes. The lower the L_p_, the stronger the information transmission capability of the network. (Shah et al., 2017). The formula:
$$L_{p}=\left[1/n(n-1)\right]\times \Sigma_{i\neq j\in G}L_{\text{ij}}$$
⑤ A small-world network means a model between a regular network and a random network. Compared with random network, the small-world network has higher C_p_ and similar L_p_, which indicates that the functional integration and differentiation of the network reach the optimal balance state. For each patient, the study generated 1,000 random networks, all of which have the the same nodes and degrees. The small-world property is measured by σ = λ/γ, if the brain network has the small world attribute, the following conditions should be met: The normalized clustering coefficients ≫1 (γ = C_p_/Crandom≫1); The normalized clustering coefficients ≈1 (λ = L_p_/Lrandom≈1); The small-world property>1(σ = λ/γ > 1). Crandom is the random network’s clustering coefficient; Lrandom is the random network’s characteristic path length.
⑥ Node degree centrality (K_i_) is the number of all edges that node i shares with other nodes in the network, which indicates the importance of a single node. The formula:
Ki=1/n∑ki
(2) Edge analysis: *T*-tests were performed on the edges of both groups with NBS (Network-Based Statistic) correction method, setting the edge *p* < 0.01 and the component *p* < 0.05, and the number of permutations was 5,000 times. In order to further locate the changes in the WM structural connection strength in specific areas of brain, with the NBS (Network-Based Statistic) used. NBS methodology was described by Zalesky et al. (2010).

### 2.6. Rich-club analysis

Referring to the article describing the organization of the rich club in brain (Grayson et al., 2014), the FN brain network is accurately tracked for all subjects’ DTIs, and more than 50% of the fiber connections in the subjects are retained to build a binary group average network (that is, the connections in more than 50% of the subjects are set as one, and vice set as zero). Calculate the rich-club coefficient of the group average network. If phi_ norm > 1, indicating that rich-club structure remains in the network and can be analyzed.

This study defines hub nodes by node degree. When the node degree value of a brain region is greater than the average ± standard deviation of all nodes in the network, this node is called the core node. Calculate the nodal degree value. The nodes whose degree value is higher than the network average degree value are called rich nodes, and the other nodes are called non-rich nodes. Therefore, the connecting edges can be divided into three categories: the connections between rich nodes, called rich connections; the connection between non-rich nodes, called local connection; the connection between rich nodes and non-rich nodes, called feeder connection. Calculate three kinds of connections of all subjects and make statistical comparison between groups. Finally, use the BrainNet Viewer toolbox (website: http://www.nitrc.org/projects/bnv/) visualizing the brain regions of rich-club.

### 2.7. Statistical analysis

All data were analyzed by IBM SPSS statistics (version 22). For continuous variables, satisfying a normal distribution, the mean ± standard deviation was used. The median (interquartile spacing) was used if the normal distribution was not satisfied, and for categorical variables, *N* (%) was used. For comparative analysis between groups, independent sample *t*-test was used for continuous variables satisfying normal distribution and chi-square test was used for categorical variables. The correlation analysis between Rich club network parameters and MoCA scores of each group was conducted by Pearson after controlling gender and age. When *p* < 0.05, the difference was statistically significant.

## 3. Results

### 3.1. Baseline characteristics of the participants

Sixty subjects were included in the study. Blood pressure measurement, blood testing, echocardiography and MoCA scale tests (summarized in Table 1B) were performed in each subjects. No significant differences were observed in age, gender, educational level, marital status, BMI, smoke, duration of HF, NYHA class, Medical history, heart rate, LEVF, heart rate, Hematochemical data between the CHF patients with CI and controls (*p* > 0.05; Table 1A). The MoCA scale scores of these two groups were compared. The results demonstrated that the total score of CHF + CI group was lower than the controls significantly (*p* < 0.001). Among them, the score of the CHF + CI group in Visuopathial/Executive function, Attention/Calculation and Orientation was lower than the controls (*p* < 0.05), but no significant difference in naming, language, abstraction and delayed recall between the CHF + CI group and the CHF group (Table 1B). The details are summarized in Table 1. Both groups of participants were right-handed.

**Table 1:** All data are expressed as mean (SD) [range].

Variables	CHF (*n* = 30)	CHF + CI (*n* = 30)	*t*/*x*^2^ value	Value of *p*
*(A) Clinical and demographic details [N (%)]*				
Baseline demographics				
Age, y, mean (SD) [range]	75(3.5)[69–81]	74(3.3)[70–81]	0.831	0.409
Sex, number of females (percentage)	14(46.67%)	16(53.33%)	−0.509	0.613
Education, high School or above (percentage)	21(70.00%)	22(73.33%)	−0.282	0.779
Marital Status, married (percentage)	27(90.00%)	25(83.33%)	−0.750	0.456
BMI, mean (SD) [range]	27.17(4.8)[19–36]	29.20(4.1)[20–36]	−1.772	0.082
Smokers, number (percentage)	11(36.67%)	9(30.00%)	−0.540	0.591
Medical history at the first visit				
Duration of heart failure, y, mean (SD) [range]	2.87(1.3)[1–6]	2.93(1.5)[1–6]	−0.182	0.875
Hypertension, number (percentage)	25(83.33%)	26(86.67%)	−0.356	0.723
Diabetes mellitus, number (percentage)	13(43.33%)	12(40.00%)	0.513	0.610
Atrial fibrillation, number (percentage)	7(23.33%)	9(30%)	−0.576	0.567
NYHA class				
I, number (percentage)	4(13.33%)	3(10.00%)	−0.396	0.694
II, number (percentage)	12(40.00%)	13(43.33%)	0.258	0.798
III, number (percentage)	10(33.33%)	11(36.67%)	0.266	0.791
IV, number (percentage)	4(13.33%)	3(10.00%)	−0.396	0.694
Hematochemical data				
NT-proBNP, pg./mL, mean (SD) [range]	475.93(312.8)[105–1,320]	444.90(355.4)[120–1,400]	0.359	0.721
LDL, mmol/L, mean (SD) [range]	2.68(0.72)[1.72–4.20]	2.61(0.75)[1.67–4.20]	0.365	0.717
HDL, mmol/L, mean (SD) [range]	1.17(0.14)[0.98–1.65]	1.21(0.13)[1.01–1.42]	−1.395	0.168
Serum creatinine, umol/L, mean (SD) [range]	67.14(19.90)[22.1–100.5]	67.80(13.07)[37.2–96.2]	−0.152	0.879
Serum urea, mmol/L, mean (SD) [range]	5.64(1.20)[3.15–8.41]	6.26(1.78)[3.00–9.93]	−1.536	0.130
Heart rate, beats/min, mean (SD) [range]	72.60(11.19)[53–88]	69.77(10.12)[55–87]	1.029	0.308
LVEF, %, mean (SD) [range]	57.66(4.07.53)[35–70]	58.83(5.48)[42–68]	−0.686	0.496
*(B) MoCA scale test details [N (%)]*				
Variables	HF (*n* = 30)	HF + CI (*n* = 30)	t/x^2^ value	value of p
MoCA total score, score, mean (SD) [range]	26.63(1.13)[26–30]	24.10(1.21)[20–25]	8.372	<0.001
Visuospatial/Executive, score, mean (SD) [range]	4.30(0.88)[2–5]	3.60(1.30)[1–5]	2.442	0.018
Naming, score, mean (SD) [range]	2.77(0.43)[2–3]	2.60(0.67)[1–3]	1.141	0.259
Attention/calculation, score, mean (SD) [range]	5.17(0.91)[3–6]	4.37(1.43)[1–6]	2.588	0.012
Language, score, mean (SD) [range]	2.83(0.38)[2–3]	2.80(0.41)[2–3]	0.328	0.744
Abstraction, score, mean (SD) [range]	1.90(0.31)[1–2]	1.83(0.38)[1–2]	0.750	0.456
Delayed recall, score, mean (SD) [range]	4.43(0.77)[3–5]	4.17(0.99) [2–5]	1.166	0.249
Orientation, score, mean (SD) [range]	5.23(0.77)[4–6]	4.73(1.08)[3–6]	2.060	0.044

SD, standard deviation; BMI, body mass index; NT-proBNP, N terminal pro B type natriuretic peptide, LDL, the low density lipoprotein; HDL, the high density lipoprotein; NYHA, the New York heart association; LVEF, the left ventricular ejection fraction; MoCA, Montreal cognitive assessment.

### 3.2. Comparison of global network properties

**Figure 1:**
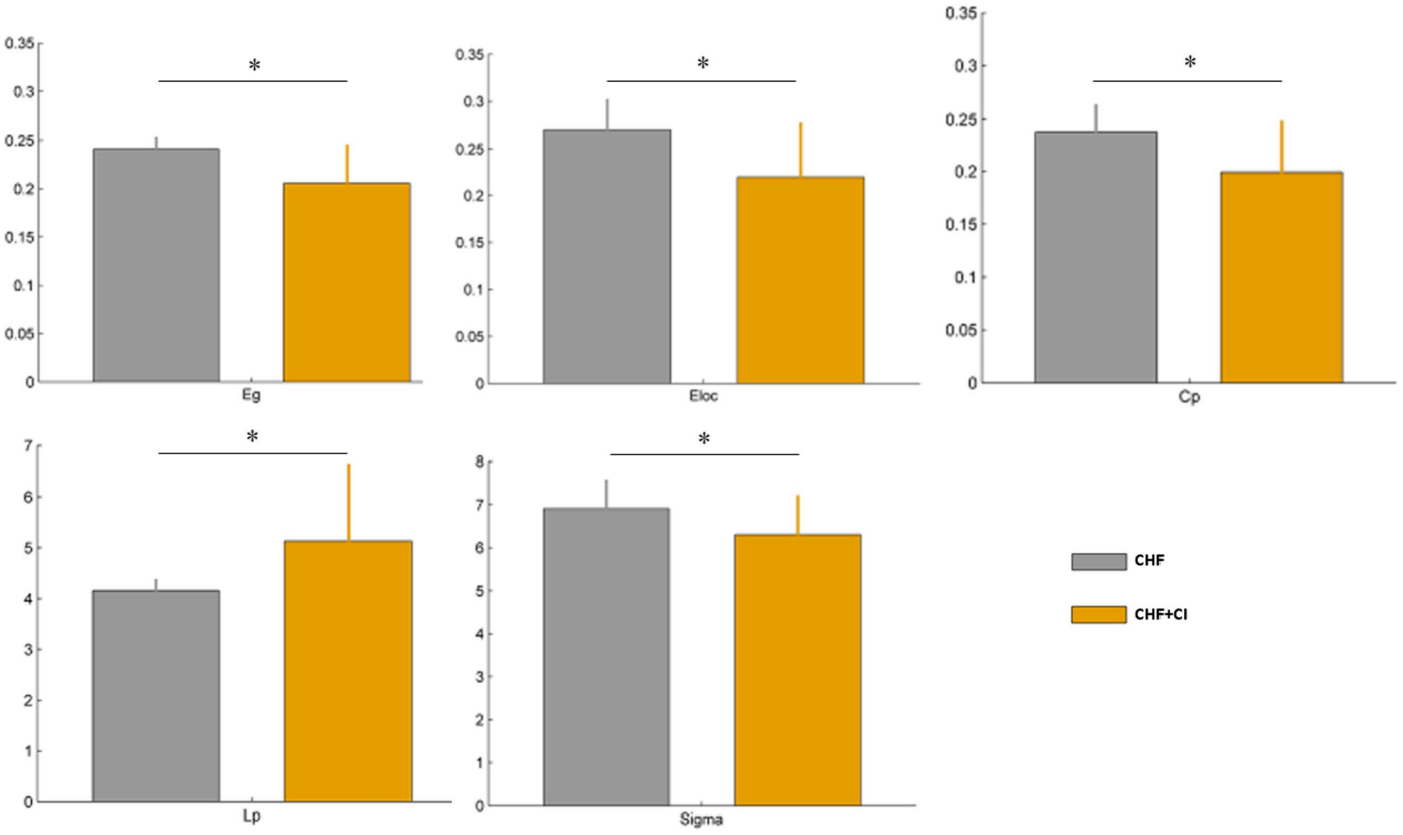
The differences in the global network measure subcortical structures between two groups. The global efficiency (Eglobal, Eg), the local efficiency (Elocal, Eloc), the clustering coefficient (Cp), the small-world attribute (sigma, σ) were significantly reduced in the CHF patients complicated with CI (CHF + CI) compared with the controls (CHF). The shortest path length (Lp) was longer in the CHF + CI group than in the CHF group (*p < 0.05, FEW corrected).

Compared with controls, CHF + CI group had a decrease in global efficiency, local efficiency, clustering coefficient, the small-world attribute, and an increase in the shortest path length (p<0.05, FEW corrected; Figure 1).

### 3.3. Comparison of node properties

**Figure 2:**
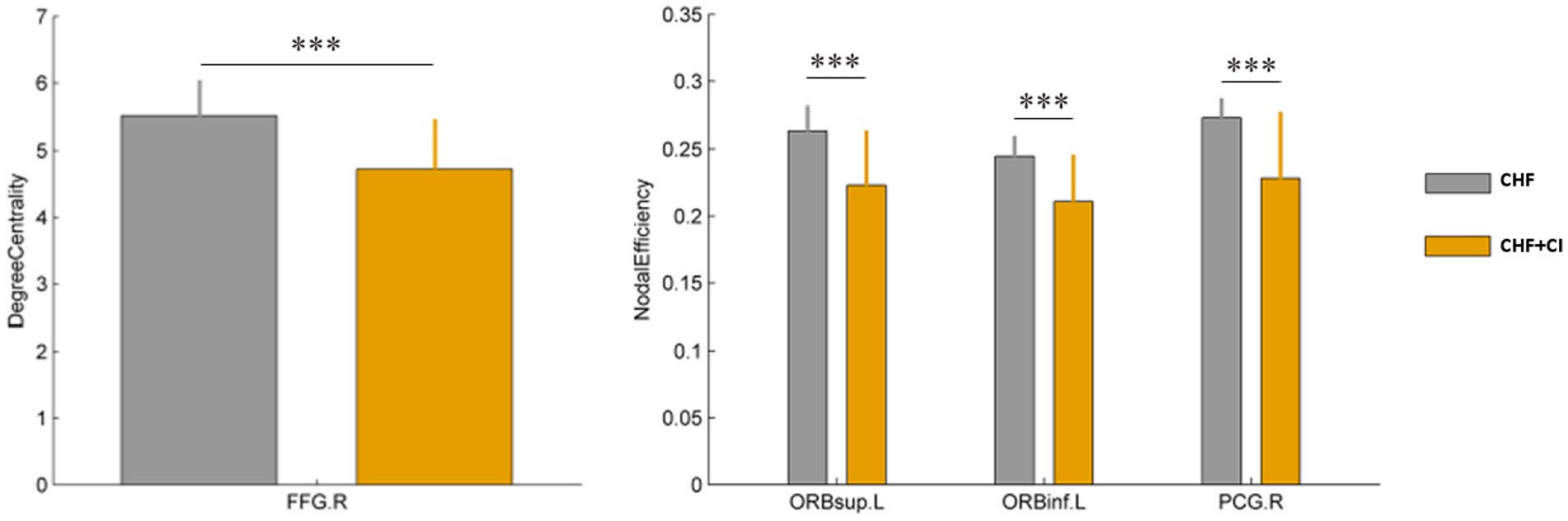
Differences in the local network measures of subcortical structures between two groups, including nodal degree centrality and nodal efficiency (***p < 0.001, FEW corrected).

The CHF + CI group showed a decrease of nodal degree centrality in the right fusiform gyrus, compared with the controls, (FFG.R; *p* < 0.001, FEW corrected); Moreover, a reduced nodal efficiency observed in the left orbital superior frontal gyrus (ORB sup. L), the left orbital inferior frontal gyrus (ORB inf. L), and the right posterior cingulate gyrus (PCG.R) in the CHF + CI group compared with controls (*p* < 0.001, FEW corrected; Figure 2).

### 3.4. Edge analysis

**Figure 3:**
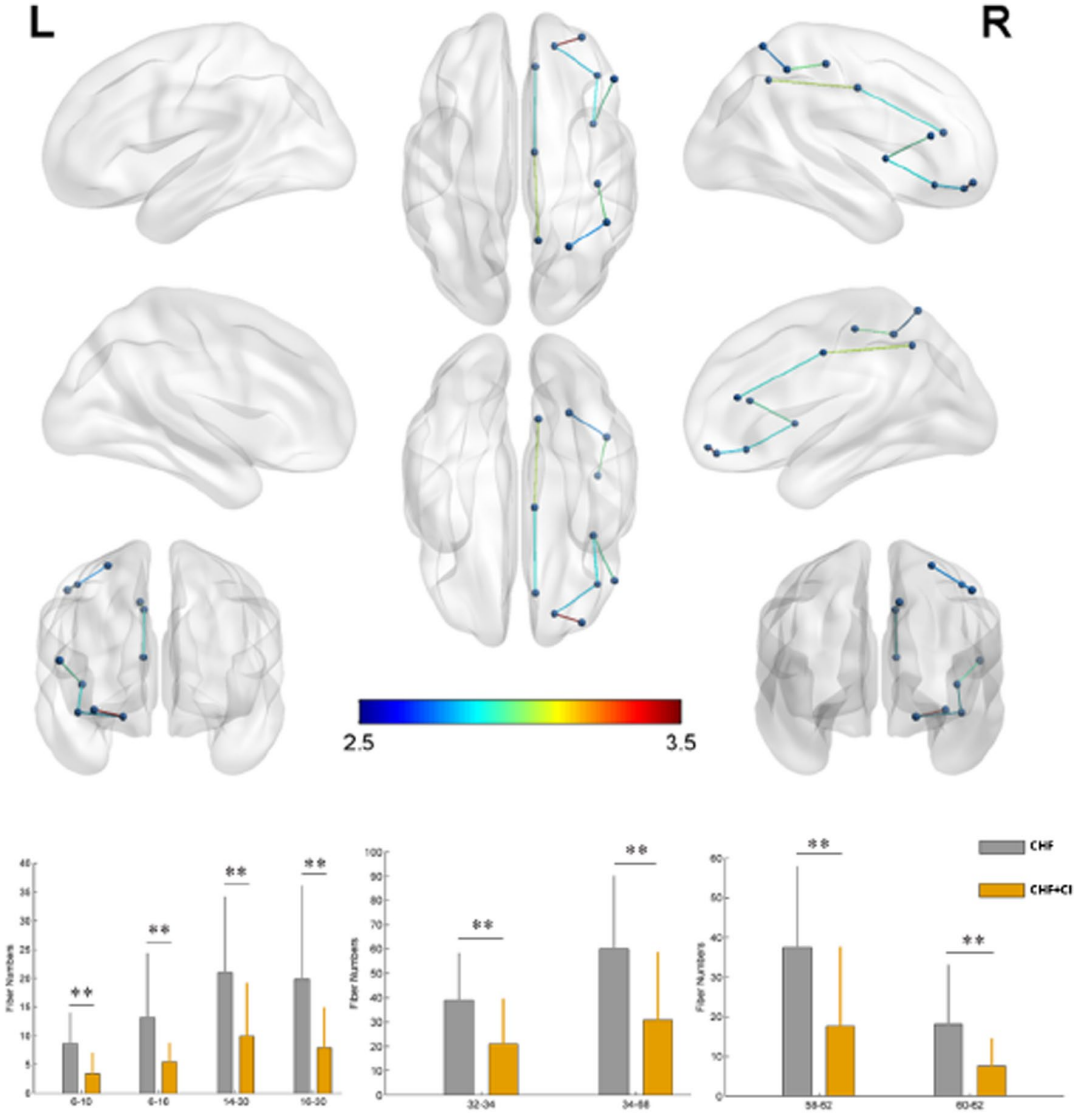
Connection comparisons between the CHF group and the CHF + CI group. Regions exhibited significantly decreased connectivity in the CHF + CI compared to the CHF (NBS corrected **p < 0.01).

The edge analysis was performed on the fiber number. The NBS approach identified three connected components of reduced connectivity in the CHF patients with CI (Figure 3). The first connected component contains the following four connected edges (the component p<0.001), the orbital superior frontal gyrus (ORB sup. R, 6) to the orbital middle frontal gyrus (ORB mid. R, 10) on the right, the orbital superior frontal gyrus (ORB sup. R, 6) to the orbital inferior frontal gyrus (ORB inf. R, 16) on the right, the triangular inferior frontal gyrus (IFG triang, R, 14) to the insula (INS.R, 30) on the right, the orbital inferior frontal gyrus (ORB inf. R, 16) to the insula (INS.R, 30) on the right. The second connected component contains the following two connected edges (the component *p* < 0.05), the anterior cingulate and paracingulate gyri (ACG.R,32) to the median cingulate and paracingulate gyri (DCG.R,34) on the right, the median cingulate and paracingulate gyri (DCG.R,34) to the precuneus (PCUN.R,68) on the right. The third connected component contains the following two connected edges (the component *p* < 0.05), the postcentral gyrus (PoCG.R,58) to the inferior parietal, but supramarginal and angular gyri (IPL.R,62) on the right, the superior parietal gyrus (SPG.R,60) to the inferior parietal, but supramarginal and angular gyri (IPL.R,62) on the right. Compared with the controls, the fiber number in the CHF patients with CI reduced, and the connecting lines decreased (Figure 3).

### 3.5. Association between global properties of brain network, edge analysis and MoCA scale

**Figure 4:**
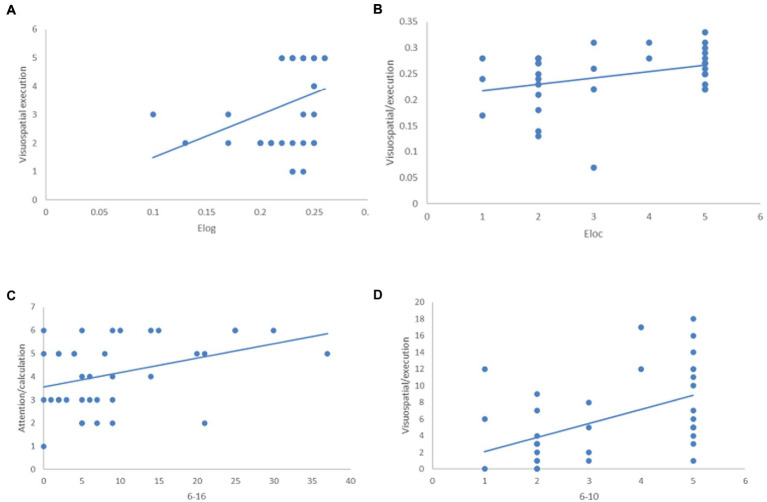
The relationship between global properties of brain network, edge analysis and MoCA scale. In CHF + CI group, Elog, Eloc and 6–10 are positively correlated with Visuospatial/Executive function **(A)** *p* = 0.042; **(B)** *p* = 0.031; **(C)** *p* = 0.002; 6–16 is positively correlated with attention/calculation **(D)**, *p* = 0.020.

In CHF patients with CI, global efficiency, local efficiency and the connected edge of the orbital superior frontal gyrus (ORB sup. R, 6) to the orbital middle frontal gyrus (ORB mid. R, 10) on the right were positively correlated to Visuospatial/Executive function (A, *p* = 0.042; B, *p* = 0.031; C, *p* = 0.002). The connected edge of the orbital superior frontal gyrus (ORB sup. R, 6) to the orbital inferior frontal gyrus (ORB inf. R, 16) on the right is positively related to attention/calculation (C, *p* = 0.020; Figure 4).

### 3.6. Rich-club analysis

Calculate the rich-club coefficient of the group average network (A). Calculate each nodal degree values of the group average network. According to the above definition method, we found that the core node is 6, 43, 44, 50, 67, 68, 73, 74, 83, 84, 85, a total of 11 brain regions in the AAL90. Their degree values are higher than the standard deviation of the network average degree values (the average degree value of the group average network is 6.67, and the standard deviation is 3.32). The 11 brain regions were defined as rich-club brain regions (B, red nodes), and the remaining 79 brain regions were defined as non rich-club brain regions. The 11 rich-club brain regions were including the right orbital superior frontal gyrus (ORB. R, 6); the left calcarine fissure and surrounding cortex (CAL. L, 43); the right calcarine fissure and surrounding cortex (CAL. R, 44); the right superior occipital gyrus (SOG. R, 50); the left precuneus (PCUN. L, 67); the right precuneus (PCUN. R, 68); the left lenticular nucleus, putamen (PUT. L, 73); the right lenticular nucleus, putamen (PUT. R, 74); the left temporal pole: superior temporal gyrus (TPOsup. L, 83); the right Temporal pole: superior temporal gyrus (TPOsup. R, 83); the left middle temporal gyrus (MTG. L, 85).

**Figure 5:**
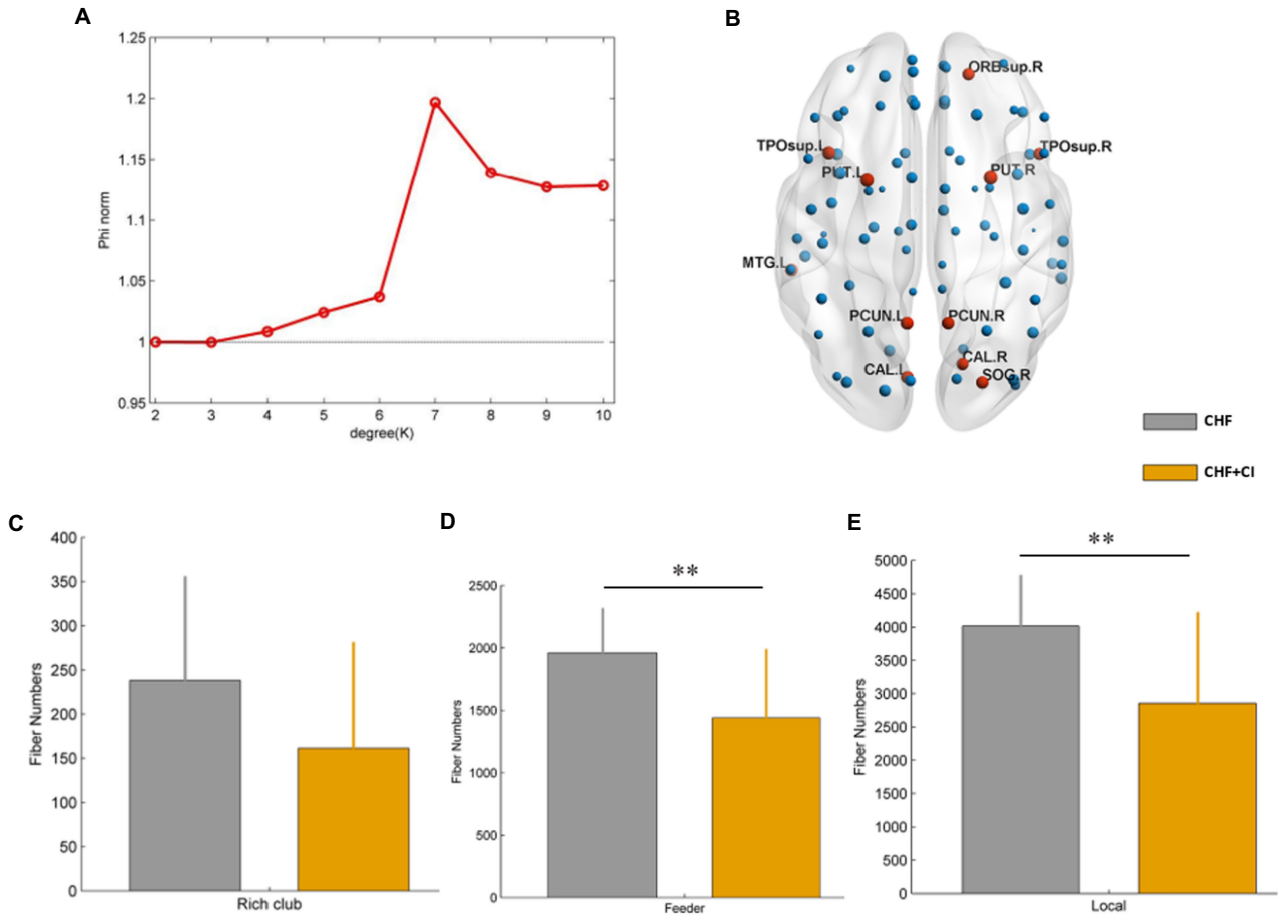
The rich-club coefficient of the group average network is calculated **(A)**. The brain regions were selected as rich-club brain regions (red nodes) and non rich-club brain regions (blue nodes). **(B)** No significant difference in rich-club connection strength between the CHF group and the CHF + CI group. **(C)** The connection strength of feeder connection (*p* < 0.01) and local connection (*p* < 0.01) in CHF + CI group was significantly reduced. **(D,E)** (**p < 0.01).

Calculate the rich-club, feeder and local connection of all subjects, and make statistical comparison between groups. Compared with the controls, the connection strength of feeder connection (*p* < 0.01) and local connection (*p* < 0.01) in CHF + CI group was significantly reduced (D, E; Figure 5). According to the research results, the connection strength between non rich-club nodes and rich-club nodes, and between non rich-club nodes of CHF + CI patients is weak. However, the rich-club connection strength in CHF + CI group was decreased compared with the CHF group, there was no statistical difference(C; Figure 5).

### 3.7. Association between brain network connectivity and MoCA scale

**Figure 6:**
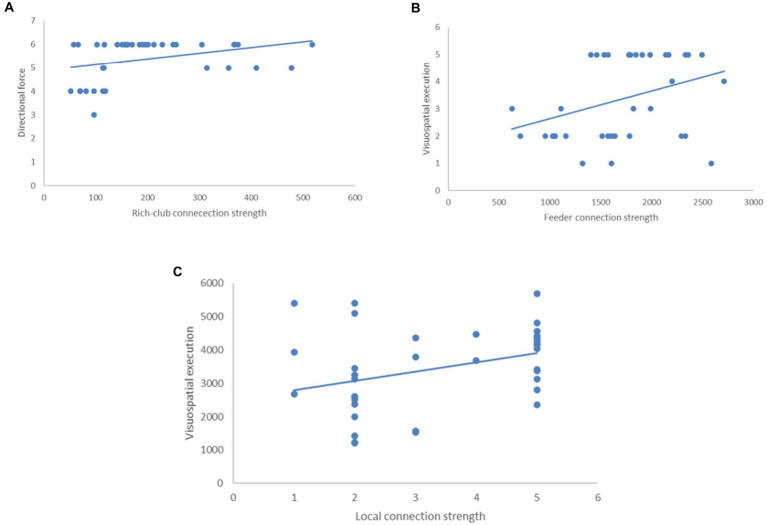
The relationship between connectivity of brain network and MoCA scale in the CHF + CI group. **(A)** The rich-club connection strength was related to the orientation (direction force; *p* = 0.043). **(B)** The feeder connection strength was related to Visuospatial/Executive function (*p* = 0.032). **(C)** The local connection strength was related to Visuospatial/Executive function (*p* = 0.032, B; *p* = 0.038).

In the CHF + CI group, the rich-club connection strength was related to the orientation (direction force) of MoCA scale (*p* = 0.043, A). The feeder and local connection strength was related to the Visuospatial/Executive function of MoCA scale (*p* = 0.032, B; *p* = 0.038, C; Figure 6).

## 4. Discussion

In recent years, the damage of chronic heart failure (CHF) to the central nervous system has attracted a lot of attention. Large scale clinical studies have clarified the relationship between CHF and cognitive impairment (CI), confirming that the disease can cause cognitive functions such as orientation, calculation, attention and executive function to decline (Alagiakrishnan et al., 2016; Fendler et al., 2021). Our study characterizes brain network properties and connectivity in CHF patients with CI. Previously evaluated by DTI-MRI, these subjects’ brain network analyzed by parameter calculation, edge analysis, rich-club analysis and correlation statistics. In this way, as compared to CHF patients without CI, we comprehensively depicted the brain network structure and interconnections of specific brain regions in CHF patients with CI, and analyzed the correlation between the brain network attributes and connectivity of the CHF patients with CI and the cognitive measures.

When analyzing the baseline data, this study found that compared with CHF patients without CI, the cognitive areas affected by CHF patients with CI mainly include Visuospatial/Executive function, Attention/Calculation and Orientation, and some detailed neuropsychological tests also revealed that affected cognitive domains encompass these above areas (Sauvé et al., 2009). We hypothesized that the impairments in different cognitive domains may be associated with the functional impairment of different brain regions in these patients.

In the comparison of global properties, we found that the brains of CHF patients with CI showed significantly smaller clustering coefficients, longer characteristic path lengths, lower local and global efficiencies, and smaller small-worldness parameters. These changes are reflective of an abnormal topology network in these patients (Hermann et al., 2021). Its significance mainly indicates that the information conversion and transmission capability among brain nodes decreases, the tightness of local network connections decreases, and the networks’ balance of functional integration and differentiation is damaged (Nemzer et al., 2020; Shigemoto et al., 2021).

In local efficiency, the damage to the connection between two nodes in the same modules will lead to a decline of specialized processing capacity (Chen et al., 2019). The decreased degree centrality of the right fusiform gyrus region demonstrates its degraded function as a hub node (Ogura et al., 2019). It is reported that the right fusiform gyrus participates in Visuospatial/Executive function, especially in character recognition (Donnelly et al., 2011). In addition, we found that the nodal efficiency is similar to the global efficiency, which reflects the damage to the neighborhood relationships between two nodes, leading to the decline of specialized processing capacity (Chen et al., 2019). The nodes involved are mainly in the left orbital superior frontal gyrus (ORB sup. L), the left orbital inferior frontal gyrus (ORB inf. L), and the right posterior cingulate gyrus (PCG.R). Research data show that the orbitofrontal cortex (OFC) and its white matter fiber connection network participate in human executive function, while the cingulate cortex is associated with visual information transfer (Nejati et al., 2018). We speculate that the decline of the nodal efficiency in the study may be related to the impairment of some cognitive domains.

The default mode network (DMN), attention network (AN), cingulo-opercular network (CON), sensory-motor network (SMN), frontoparietal network (FPN), visual network (VN) are currently recognized as the brain networks (Chen et al., 2016). According to previous neuroimaging studies, a group of interconnected brain networks, such as DMN, AN and FPN, are demonstrated to play an important role in cognitive processing (Eyler et al., 2019; Cascone et al., 2021). These brain regions are composed of different nodes, such as the orbital cortex (ORB), the inferior frontal gyrus (IFG), the anterior cingulate (ACG), the precuneus (PCUN) etc. which are connected by white matter. In our study, the number of white matter connections was significantly smaller in the CHF patients complicated with CI than in the CHF patients without CI. According to previous studies, multiple regions are involved in cognitive control related to executive function, namely, IFG, anterior insula, OFC, when their white matter connectivity declines, which was observed in the first connected component of our study, may lead to effective interference resolution behaviorally (Levens and Phelps, 2010). Similar changes were observed in the other two connected component. Some studies on mild cognitive impairment (MCI) found that functional connectivity in the right hippocampal functional networks, including the right anterior cingulate and paracingulate gyri (ACG.R) and precuneus (PCUN), was decreased (Feng et al., 2019), which was close to our analysis of the second connected component. The main components of the DMN, which is involved in visual space planning according to recent evidence, contain some of these brain regions (Vatansever et al., 2018). The AN includes part of frontal and parietal cortices. Cortical lesions involving frontal and parietal cortices and their connections can lead to attention deficits, such as visual–spatial neglect, a syndrome with the inability to direct attention to contractile spaces (Fiebelkorn and Kastner, 2020). In addition, subjects with higher spatial orientation accuracy showed greater activity in the supramarginal gyrus (Arnold et al., 2014). When the connections of these brain regions are damaged, as we analyzed in the third connected component, attention deficit and decreased accuracy of spatial orientation may occur. In addition, the left and right hemispheric networks of the subjects are damaged differently, which may be related to different degrees of cognitive impairment (Vatansever et al., 2018).

We further analyzed the correlation between the topological properties, edge connectivity and the intelligent scale. The results confirmed that the changes of global efficiency, local efficiency and white matter connectivity between the orbitofrontal lobe were positively correlated with visual space/executive function; the connection between the orbital superior frontal gyrus and the orbital inferior frontal gyrus was related to attention/calculation positively. As a flexible hub, the orbitofrontal lobe maintains homeostatic balance through multiple brain functional networks according to task requirements (Cole et al., 2014). According to previous studies, the FPN plays a role in information processing, executive function and attention (Posner et al., 2016). Our findings confirm that a correlation between cognitive impairment and functional alterations in brain networks in CHF patients with CI.

Highly interconnected hub regions of brain network often form rich-clubs (Csigi et al., 2017). The rich-club is essential as it helps to optimize cognitive processes and possess highly efficient neural information transfer (Kim and Min, 2020). In CHF patients with CI, we statistically observed decreased rich-club, local and feeder connections strength, suggesting widespread decline in brain network connectivity. The CHF patients with CI showed feeder and local connection strength decreased, whereas there was an uncertain decreased trend in rich-club connections. We speculate that the degree of cognitive impairment in CHF patients may be different, and the rich-club connections may not be significantly affected in the early stage of cognitive impairment. Due to the different connection brain regions, the cognitive function alteration caused by the strength changes of rich-club, feeder and local connections may also be different. A structural network study have shown that non-Rich-club connections are disrupted before Rich-club connections in patients with the preclinical state of Alzheimer’s disease (Yan et al., 2018). Another study also showed that the lack of significant impairment of Rich-club connections in some diseases may be due to a higher reserve capacity of the central nervous system to resist aging and disease damage (Staff, 2012). By correlation analysis, we can also find that rich-club connections is positively correlated with orientation, while feeder and local connections are positively correlated with Visuospatial/Executive power. This interesting finding explains that when analyzing the topological properties and edge connectivity of brain networks, we have observed more changes in the function and connectivity of brain regions such as FPN, which may be related to the decrease of feeder and local connections strength.

The underlying molecular mechanism of chronic heart failure with cognitive decline is still unclear, and numerous scholars are working on it. A recent study showed a correlation between NT-proBNP and neuroflament light chain in serum of chronic heart failure patients accompanied with cognitive impairment (Traub et al., 2022), which explained the correlation between heart failure and cognitive impairment at the molecular level. Another study indicated that epigenetic gene expression plays a role in the link between chronic heart failure and cognitive impairment (Islam et al., 2021). More experiments may be needed to prove this conclusion.

From the perspective of brain network attributes, our research not only proved that the topological attributes and the integrity of connections of brain networks was important in maintaining cognitive function, but also confirmed the importance of frontal orbitals, cingulate gyrus, precuneus and other brain regions in executive power, attention and other cognitive areas. There are still limitations in our research. The small sample size of CHF patients and the poor cognitive function and physical ability of a subset of patients may limit the generalizability of the study. In addition, this is a cross-sectional study, and in the future, we should try to observe and compare changes in brain networks and cognitive function at different stages of heart failure.

CHF patients complicated with cognitive impairment exhibit selective cognitive impairments in the domains of Visuospatial/Executive function, Attention/Calculation and Orientation. The application of diffusion tensor imaging technology suggests the topological attributes and the integrity of connections of brain networks maybe probable correlate with cognitive impairment in CHF patients. These technological advances are increasing rapidly. The wide application of these technologies clinically in the future provides more possibilities for predicting early assessment, delaying CI progress and improving patients’ quality of life.

## Data availability statement

The original contributions presented in the study are included in the article/supplementary material, further inquiries can be directed to the corresponding author.

## Author contributions

MW: conception and design of study, analysis and interpretation of data, and drafting the manuscript. QS and BX: conception and design of study, analysis and interpretation of data. XH, HZ, QG, GW, XD, MS, QX, and QC: acquisition of data, analysis and interpretation of data. HF: conception and design of study, drafting the manuscript, and revising the manuscript. All authors contributed to the article and approved the submitted version.

## Funding

This work was supported by Suzhou College of Nanjing Medical University (GSKY20210207); Suzhou Science and Education Youth Project (KJXW2021035); China clinical trial registration number (ChiCTR210048345), and Science and Technology Plan Project of Suzhou (No. skjyd2021223).

## Conflict of interest

The authors declare that the research was conducted in the absence of any commercial or financial relationships that could be construed as a potential conflict of interest.

The reviewer MZ declared a shared parent affiliation with the authors to the handling editor at the time of review.

## Publisher’s note

All claims expressed in this article are solely those of the authors and do not necessarily represent those of their affiliated organizations, or those of the publisher, the editors and the reviewers. Any product that may be evaluated in this article, or claim that may be made by its manufacturer, is not guaranteed or endorsed by the publisher.
